# Supplementary material for: Comparative genomics provides new insights into the diversity, physiology, and sexuality of the only industrially exploited tremellomycete: Phaffia rhodozyma
Source: BMC Genomics. 2016 Nov 9;17:901. doi: 10.1186/s12864-016-3244-7 (PMC5103461; doi:10.1186/s12864-016-3244-7)
Supplement: Additional file 6: — List of orphan genes with links to PFAM (related to Additional file 1: Table S1). (ZIP 1428 kb) [file 12864_2016_3244_MOESM6_ESM.zip › BLAST_HTML_FTR/G02917_P.html]

BLAST Search Results


```
BLASTP 2.2.27+


Reference:
Stephen F. Altschul, Thomas L. Madden, Alejandro A. Schäffer,
Jinghui Zhang, Zheng Zhang, Webb Miller, and David J. Lipman (1997),
"Gapped BLAST and PSI-BLAST: a new generation of protein database
search programs", Nucleic Acids Res. 25:3389-3402.


Reference for
composition-based statistics:
Alejandro A. Schäffer, L. Aravind, Thomas L. Madden, Sergei
Shavirin, John L. Spouge, Yuri I. Wolf, Eugene V. Koonin, and
Stephen F. Altschul (2001), "Improving the accuracy of PSI-BLAST
protein database searches with composition-based statistics and
other refinements", Nucleic Acids Res. 29:2994-3005.


Database: nr
           71,551,133 sequences; 26,053,659,533 total letters


Query= G02917_P

Length=413
                                                                      Score     E
Sequences producing significant alignments:                          (Bits)  Value

emb|CED83466.1|  cytochrome c subunit vib [Xanthophyllomyces dend...   818    0.0  
emb|CED84411.1|  hypothetical protein [Xanthophyllomyces dendrorh...   147    4e-36
gb|KKY35872.1|  hypothetical protein UCDDA912_g04144 [Diaporthe a...  49.7    0.003
gb|KIY67914.1|  hypothetical protein CYLTODRAFT_422121 [Cylindrob...  46.6    0.031
emb|CDS13734.1|  hypothetical protein LRAMOSA05908 [Absidia idaho...  43.9    0.31 
emb|CDZ97774.1|  hypothetical protein [Xanthophyllomyces dendrorh...  42.0    1.1  
gb|KOP43034.1|  Transcription elongation factor SPT6 [Madurella m...  41.6    1.4  
gb|KFH65257.1|  hypothetical protein MVEG_08738 [Mortierella vert...  41.2    2.3  
gb|KIK44320.1|  hypothetical protein CY34DRAFT_642275 [Suillus lu...  40.4    3.1  
ref|XP_007794722.1|  hypothetical protein UCREL1_6830 [Eutypa lat...  40.0    4.1  
ref|WP_022559002.1|  MULTISPECIES: hypothetical protein [Xanthomo...  40.0    4.3  
ref|WP_039731723.1|  hypothetical protein [Xanthomonas fuscans] >...  39.7    4.4  
ref|WP_024892184.1|  MULTISPECIES: hypothetical protein [Gammapro...  39.7    4.6  
ref|WP_033485019.1|  hypothetical protein [Xanthomonas campestris...  39.7    4.7  
ref|WP_033987660.1|  hypothetical protein [Pseudomonas aeruginosa]    39.7    4.9  
gb|EMS21313.1|  hypothetical protein RHTO_01779 [Rhodosporidium t...  39.7    6.2  
ref|XP_001830880.2|  hypothetical protein CC1G_02331 [Coprinopsis...  39.3    7.1  
ref|WP_009466141.1|  hypothetical protein [Roseibium sp. TrichSKD...  38.9    7.9  
ref|WP_032830587.1|  hypothetical protein [Pseudomonas sp. GM79]      38.9    8.9  
ref|XP_008040170.1|  hypothetical protein TRAVEDRAFT_49159 [Trame...  38.5    9.0  


 >emb|CED83466.1| cytochrome c subunit vib [Xanthophyllomyces dendrorhous]
Length=590

 Score =  818 bits (2112),  Expect = 0.0, Method: Compositional matrix adjust.
 Identities = 412/412 (100%), Positives = 412/412 (100%), Gaps = 0/412 (0%)

Query  1    MAFTGKIPTFIPIDIYREVIGQLFLDSISPNWLEREEIKDERRIKLFEDLLSCCLVSKEW  60
            MAFTGKIPTFIPIDIYREVIGQLFLDSISPNWLEREEIKDERRIKLFEDLLSCCLVSKEW
Sbjct  1    MAFTGKIPTFIPIDIYREVIGQLFLDSISPNWLEREEIKDERRIKLFEDLLSCCLVSKEW  60

Query  61   LAITRPFLYLDIDLSCIDNLDALFNTLSLNPYIRPYVKHLSFPMTSIELLETKRHLFPCA  120
            LAITRPFLYLDIDLSCIDNLDALFNTLSLNPYIRPYVKHLSFPMTSIELLETKRHLFPCA
Sbjct  61   LAITRPFLYLDIDLSCIDNLDALFNTLSLNPYIRPYVKHLSFPMTSIELLETKRHLFPCA  120

Query  121  QLSFPYDFDTDGSLIDGIPDSLLSFTRWERKIQLRKIVIASWQGRSWEPAARVLIRASEI  180
            QLSFPYDFDTDGSLIDGIPDSLLSFTRWERKIQLRKIVIASWQGRSWEPAARVLIRASEI
Sbjct  121  QLSFPYDFDTDGSLIDGIPDSLLSFTRWERKIQLRKIVIASWQGRSWEPAARVLIRASEI  180

Query  181  FDLTQLEALEFSELPVGDTELELAENPKSSDLLEPLRRILLDSFGPKMQELKITTTSRLL  240
            FDLTQLEALEFSELPVGDTELELAENPKSSDLLEPLRRILLDSFGPKMQELKITTTSRLL
Sbjct  181  FDLTQLEALEFSELPVGDTELELAENPKSSDLLEPLRRILLDSFGPKMQELKITTTSRLL  240

Query  241  LLLFLDSLPDLQRLSISADLANMILNRTSYPRLESLQIMTDPDIEPSPIQEHPDLLFSRN  300
            LLLFLDSLPDLQRLSISADLANMILNRTSYPRLESLQIMTDPDIEPSPIQEHPDLLFSRN
Sbjct  241  LLLFLDSLPDLQRLSISADLANMILNRTSYPRLESLQIMTDPDIEPSPIQEHPDLLFSRN  300

Query  301  DTLERLARAFPLLRVIGASPYMSEQDIYADAELIPDEDLFAELEEEDLISMEQRRDEMRE  360
            DTLERLARAFPLLRVIGASPYMSEQDIYADAELIPDEDLFAELEEEDLISMEQRRDEMRE
Sbjct  301  DTLERLARAFPLLRVIGASPYMSEQDIYADAELIPDEDLFAELEEEDLISMEQRRDEMRE  360

Query  361  CAVELKEVGVTLVDQWGVEWLKKPKSGLSSDWAKDILVALTDRQDGERESAM  412
            CAVELKEVGVTLVDQWGVEWLKKPKSGLSSDWAKDILVALTDRQDGERESAM
Sbjct  361  CAVELKEVGVTLVDQWGVEWLKKPKSGLSSDWAKDILVALTDRQDGERESAM  412


>emb|CED84411.1| hypothetical protein [Xanthophyllomyces dendrorhous]
Length=383

 Score =  147 bits (370),  Expect = 4e-36, Method: Compositional matrix adjust.
 Identities = 107/350 (31%), Positives = 170/350 (49%), Gaps = 38/350 (11%)

Query  15   IYREVIGQLFLDSISPNWLEREEIKDERRIKLFEDLLSCCLVSKEWLAITRPFLYLDIDL  74
            IY++++    LD  S  + +    K+ER+  +++DL    LVS+EW  +  P LY DIDL
Sbjct  9    IYQKIVTYRHLDLYS-TYRDDPSTKEERQRAVYQDLAKFSLVSREWWNMITPLLYSDIDL  67

Query  75   SCIDNLDALFNTLSLNPYIRPYVKHLSFPMTSIELLETKRHLFPCAQLSFPY--------  126
              +D +D  F  +   P     VKH+S P    +L+  +   FP  Q+S           
Sbjct  68   MAVD-IDNFFGAIDSKPSFGSLVKHISLPSNCYDLVGDREDYFPNLQISIGLEYTPGFGD  126

Query  127  -DFDTD------------GSLIDGIPDSLLSFTRWERKIQLRKIVIASWQGRSWEPAARV  173
             D++ D            G+ + G+ + +L FT WER+I +R + + S +   W+   R+
Sbjct  127  DDYEADAEKGEKEDEAQIGADLRGLEEEVLCFTDWERRISIRNLEVLSAESDGWQSVTRL  186

Query  174  LIRASEIFDLTQLEALEFSEL-----PVGDTELELAENPKSSDLLEPLRRILLDSFGPKM  228
            L     IFDL+ LE L F E+     PV  T ++L+  P    L     R+LL      +
Sbjct  187  LFSLRTIFDLSSLEGLTFIEISDPESPVSPT-IDLSAVPMFDHL-----RVLLTELSLTV  240

Query  229  QELKITTTSRLLLLLFLDSLPDLQRLSISADLANMILNRTSYPRLESLQIMTDPDIEPSP  288
            +EL +TT S  L  LF  +L +  R+SI + LA+++L +  +P + SL I T+P +EP  
Sbjct  241  RELSLTTPSIDLFRLFTRTLSNAYRMSIPSTLASLVLAQECHPTVTSLWITTEPGLEPQD  300

Query  289  IQEHPDLLFSRNDTLERLARAFPLLRVIGASPYMS----EQDIYADAELI  334
                P    +     +RL +AFP L  I   P +S    E++I  D E +
Sbjct  301  SFPRPQDFLADTKIQDRLRQAFPKLEEISTDPIVSESATEEEIKKDGEAV  350


>gb|KKY35872.1| hypothetical protein UCDDA912_g04144 [Diaporthe ampelina]
Length=325

 Score = 49.7 bits (117),  Expect = 0.003, Method: Compositional matrix adjust.
 Identities = 31/104 (30%), Positives = 51/104 (49%), Gaps = 8/104 (8%)

Query  11   IPIDIYREVIGQLFLDSISPNWLEREEIKDERRIKLFEDLLSCCLVSKEWLAITRPFLYL  70
            +P ++   +I +L  D     W  R   +D        DL S CLVSK    ITRP+LY 
Sbjct  51   LPDELLTPIIKELVYDENRSGWSARFAARD--------DLRSLCLVSKRIDLITRPYLYK  102

Query  71   DIDLSCIDNLDALFNTLSLNPYIRPYVKHLSFPMTSIELLETKR  114
             + +SC D+L  L  T+S +P++   ++ +   ++      +KR
Sbjct  103  KVFISCFDSLAKLHRTISRDPHLGDEIREIDLGVSFGSATFSKR  146


>gb|KIY67914.1| hypothetical protein CYLTODRAFT_422121 [Cylindrobasidium torrendii 
FP15055 ss-10]
Length=386

 Score = 46.6 bits (109),  Expect = 0.031, Method: Compositional matrix adjust.
 Identities = 25/58 (43%), Positives = 31/58 (53%), Gaps = 0/58 (0%)

Query  50   LLSCCLVSKEWLAITRPFLYLDIDLSCIDNLDALFNTLSLNPYIRPYVKHLSFPMTSI  107
            L SC LVSK WL +TRPF++  + L       A    L+ NP IRP V+ L    T I
Sbjct  29   LQSCQLVSKAWLQLTRPFVHEKVLLRSASQAVAFLGVLAQNPAIRPCVRTLEISETRI  86


>emb|CDS13734.1| hypothetical protein LRAMOSA05908 [Absidia idahoensis var. thermophila]
Length=569

 Score = 43.9 bits (102),  Expect = 0.31, Method: Compositional matrix adjust.
 Identities = 30/91 (33%), Positives = 42/91 (46%), Gaps = 21/91 (23%)

Query  11   IPIDIYREVIGQLFLDSISPNWLEREEIKDERRIKLFEDLLSCCLVSKEWLAITRPFLYL  70
            +PI+I R+V+  LF+DS                    ED  +C  V + W +ITRP LY 
Sbjct  3    LPIEIIRQVL--LFIDSA-------------------EDKCTCLTVCRLWYSITRPLLYK  41

Query  71   DIDLSCIDNLDALFNTLSLNPYIRPYVKHLS  101
             + L   D LD   +TL   P    YV+ L+
Sbjct  42   HVSLQSWDQLDQFVSTLVSAPTYGEYVRELA  72


>emb|CDZ97774.1| hypothetical protein [Xanthophyllomyces dendrorhous]
Length=425

 Score = 42.0 bits (97),  Expect = 1.1, Method: Compositional matrix adjust.
 Identities = 27/101 (27%), Positives = 50/101 (50%), Gaps = 8/101 (8%)

Query  4    TGKIPTFIPIDIYREVIGQLFLDSI--SPNWLEREEIKDERRIKLFEDLLSCCLVSKEWL  61
            TG  P  +P+++ R    Q+ +DS+  + ++  ++     RR   F  L+  C+VS +W 
Sbjct  20   TGTFPVTLPLEVQR----QIMIDSLDRTSSYCPKDNSAVLRRNARF--LVKMCMVSHDWK  73

Query  62   AITRPFLYLDIDLSCIDNLDALFNTLSLNPYIRPYVKHLSF  102
             +  P LY  I L  +  L    NT+  +P++  Y  +L +
Sbjct  74   DMIYPILYQRICLPRLKQLFRFMNTICASPWLGEYTVYLHY  114


>gb|KOP43034.1| Transcription elongation factor SPT6 [Madurella mycetomatis]
Length=593

 Score = 41.6 bits (96),  Expect = 1.4, Method: Compositional matrix adjust.
 Identities = 30/104 (29%), Positives = 53/104 (51%), Gaps = 16/104 (15%)

Query  11   IPIDIYREVIGQLFLDSISP-------NWLEREEIKDERRIKLFEDLLSCCLVSKEWLAI  63
            +P +I+  ++  LF   +SP        W     ++  RR    ++L    LVS+ W  +
Sbjct  141  LPAEIHECILDHLFGFRVSPASKSSITRW--GTALRHPRR----KELSGLSLVSRAWRVL  194

Query  64   TRPFLYLDIDL-SCIDNLDALFNTLSLNPYIRPYVKHLS--FPM  104
             +  LY  I L + +D+L   F+  + +P++RPYVKH+   FP+
Sbjct  195  IQERLYRHIKLKATVDSLRESFSYFTNHPHLRPYVKHIEIWFPV  238


>gb|KFH65257.1| hypothetical protein MVEG_08738 [Mortierella verticillata NRRL 
6337]
Length=698

 Score = 41.2 bits (95),  Expect = 2.3, Method: Compositional matrix adjust.
 Identities = 23/49 (47%), Positives = 27/49 (55%), Gaps = 10/49 (20%)

Query  46  LFEDLLSCCLVSKEWLAITRPFLYLDI--------DLSCIDNLDALFNT  86
           LFEDLL+C LVS+ W  +  P LYLD+        DL C  N    FNT
Sbjct  19  LFEDLLNCVLVSRAWHDVLSPILYLDVVTFRSTKSDLGC--NYIPYFNT  65


>gb|KIK44320.1| hypothetical protein CY34DRAFT_642275 [Suillus luteus UH-Slu-Lm8-n1]
Length=432

 Score = 40.4 bits (93),  Expect = 3.1, Method: Compositional matrix adjust.
 Identities = 24/60 (40%), Positives = 32/60 (53%), Gaps = 2/60 (3%)

Query  28   ISPNWLEREEIKDERRIKLFEDLLSCCLVSKEWLAITRPFLYLDIDLSCIDNLDALFNTL  87
            + P W  RE+   E   K+   LLSC LVS+ W    R  LY DI LS +++L  L  T+
Sbjct  85   LQPRWGRRED--REAWKKIVSVLLSCSLVSRAWYHAARAVLYQDIRLSQVNSLRLLLCTV  142


>ref|XP_007794722.1| hypothetical protein UCREL1_6830 [Eutypa lata UCREL1]
 gb|EMR66206.1| hypothetical protein UCREL1_6830 [Eutypa lata UCREL1]
Length=596

 Score = 40.0 bits (92),  Expect = 4.1, Method: Compositional matrix adjust.
 Identities = 30/99 (30%), Positives = 51/99 (52%), Gaps = 6/99 (6%)

Query  6    KIPTFIPIDIYREVIGQLFLDSISPNWLEREEIKDERRIKLFEDLLSCCLVSKEWLAITR  65
              PT + + I++ ++ +      +PN L+ E I  +RR      LL  CLVSK +  I +
Sbjct  6    SFPTELLLMIFQHLVPEPLPTKPTPNLLDDEYI--QRR----NALLDLCLVSKRFSHIAQ  59

Query  66   PFLYLDIDLSCIDNLDALFNTLSLNPYIRPYVKHLSFPM  104
            P L+ +I ++    L ALF+T   +P  R Y +  + P+
Sbjct  60   PLLHHNITITDGAQLVALFHTFWNSPDFRRYPRAFACPI  98


>ref|WP_022559002.1| MULTISPECIES: hypothetical protein [Xanthomonas]
 emb|CDF61463.1| Conserved hypothetical protein [Xanthomonas fuscans subsp. fuscans]
 gb|KGK64119.1| hypothetical protein NB99_21330 [Xanthomonas fuscans subsp. fuscans]
 11 more sequence titles

gb|KGP21543.1| hypothetical protein NY65_21190 [Xanthomonas axonopodis pv. phaseoli]
 gb|KGP22712.1| hypothetical protein NY67_19010 [Xanthomonas fuscans subsp. fuscans]
 gb|KGP22805.1| hypothetical protein NY68_19035 [Xanthomonas fuscans subsp. fuscans]
 gb|KGP33809.1| hypothetical protein NY64_18820 [Xanthomonas fuscans subsp. fuscans]
 gb|KGT56108.1| hypothetical protein NY96_08270 [Xanthomonas fuscans subsp. fuscans]
 gb|KGU41563.1| hypothetical protein NY95_13145 [Xanthomonas fuscans subsp. fuscans]
 gb|KGU42021.1| hypothetical protein NY97_17475 [Xanthomonas fuscans subsp. fuscans]
 gb|KGU45537.1| hypothetical protein NY94_08420 [Xanthomonas axonopodis pv. phaseoli]
 gb|KGU51685.1| hypothetical protein NY98_14960 [Xanthomonas fuscans subsp. fuscans]
 gb|KHF71706.1| hypothetical protein NY63_19960 [Xanthomonas fuscans subsp. fuscans]
 gb|KIJ03892.1| hypothetical protein ST33_01925 [Xanthomonas fuscans subsp. fuscans]

Length=358

 Score = 40.0 bits (92),  Expect = 4.3, Method: Compositional matrix adjust.
 Identities = 52/221 (24%), Positives = 96/221 (43%), Gaps = 31/221 (14%)

Query  98   KHLSFPMT--SIELLETK----RHLFPCAQLSFPYDFDTDG----SLIDGIPDSLLSFTR  147
            K + F +T  S+ELL  K      + P  Q  + ++ D       SLI G+P   L F  
Sbjct  28   KRIEFYLTEYSVELLANKMSKDEFVVPAYQREYTWEHDRKSRFIESLILGLPIPFLFF--  85

Query  148  WERKIQLRKIVIASWQGRSWEPAARVLIRASEIFDLTQLEALEFSELPVGDTELELAENP  207
            WE      +IV  S + RS E      +R  E+  LT L   +FS+LP         E+ 
Sbjct  86   WEMPNGKLEIVDGSQRLRSIEEFVLGDLRLGELQSLTALSGFKFSDLP---------ESR  136

Query  208  KSSDLLEPLRRILLDSFGPKMQEL----KITTTSRLLLLLFLDSLPDLQRLSISADLANM  263
            +       +R I+L+    +        +I T S++       ++ +++R +++    ++
Sbjct  137  QRKINNRSIRGIVLNEHADEQARFDMFERINTGSKIA------NMAEVRRGALAGPFMDL  190

Query  264  ILNRTSYPRLESLQIMTDPDIEPSPIQEHPDLLFSRNDTLE  304
            I+  +  P+  +L  ++  D++    +E     F+ +D LE
Sbjct  191  IIELSVLPKFVALAPVSKKDLDEREHEELVARFFAYSDGLE  231


>ref|WP_039731723.1| hypothetical protein [Xanthomonas fuscans]
 gb|KIF09568.1| hypothetical protein SP73_12225 [Xanthomonas fuscans subsp. fuscans]
Length=358

 Score = 39.7 bits (91),  Expect = 4.4, Method: Compositional matrix adjust.
 Identities = 52/221 (24%), Positives = 96/221 (43%), Gaps = 31/221 (14%)

Query  98   KHLSFPMT--SIELLETK----RHLFPCAQLSFPYDFDTDG----SLIDGIPDSLLSFTR  147
            K + F +T  S+ELL  K      + P  Q  + ++ D       SLI G+P   L F  
Sbjct  28   KRIEFYLTEYSVELLANKMSKDEFVVPAYQREYTWEHDRKSRFIESLILGLPIPFLFF--  85

Query  148  WERKIQLRKIVIASWQGRSWEPAARVLIRASEIFDLTQLEALEFSELPVGDTELELAENP  207
            WE      +IV  S + RS E      +R  E+  LT L   +FS+LP         E+ 
Sbjct  86   WEMPNGKLEIVDGSQRLRSIEEFVLGDLRLGELQSLTALSGFKFSDLP---------ESR  136

Query  208  KSSDLLEPLRRILLDSFGPKMQEL----KITTTSRLLLLLFLDSLPDLQRLSISADLANM  263
            +       +R I+L+    +        +I T S++       ++ +++R +++    ++
Sbjct  137  QRKINNRSIRGIVLNEHADEQARFDMFERINTGSKIA------NMAEVRRGALAGPFMDL  190

Query  264  ILNRTSYPRLESLQIMTDPDIEPSPIQEHPDLLFSRNDTLE  304
            I+  +  P+  +L  ++  D++    +E     F+ +D LE
Sbjct  191  IIELSVLPKFVALAPVSKKDLDEREHEELVARFFAYSDGLE  231


>ref|WP_024892184.1| MULTISPECIES: hypothetical protein [Gammaproteobacteria]
Length=358

 Score = 39.7 bits (91),  Expect = 4.6, Method: Compositional matrix adjust.
 Identities = 52/221 (24%), Positives = 96/221 (43%), Gaps = 31/221 (14%)

Query  98   KHLSFPMT--SIELLETK----RHLFPCAQLSFPYDFDTDG----SLIDGIPDSLLSFTR  147
            K + F +T  S+ELL  K      + P  Q  + ++ D       SLI G+P   L F  
Sbjct  28   KRIEFYLTEYSVELLANKMSKGEFVVPAYQREYTWEHDRKSRFIESLILGLPIPFLFF--  85

Query  148  WERKIQLRKIVIASWQGRSWEPAARVLIRASEIFDLTQLEALEFSELPVGDTELELAENP  207
            WE      +IV  S + RS E      +R  E+  LT L   +FS+LP         E+ 
Sbjct  86   WEMPNGKLEIVDGSQRLRSIEEFVLGDLRLGELQSLTALSGFKFSDLP---------ESR  136

Query  208  KSSDLLEPLRRILLDSFGPKMQEL----KITTTSRLLLLLFLDSLPDLQRLSISADLANM  263
            +       +R I+L+    +        +I T S++       ++ +++R +++    ++
Sbjct  137  QRKINNRSIRGIVLNEHADEQARFDMFERINTGSKIA------NMAEVRRGALAGPFMDL  190

Query  264  ILNRTSYPRLESLQIMTDPDIEPSPIQEHPDLLFSRNDTLE  304
            I+  +  P+  +L  ++  D++    +E     F+ +D LE
Sbjct  191  IIELSVLPKFVALAPVSKKDLDEREHEELVARFFAYSDGLE  231


>ref|WP_033485019.1| hypothetical protein [Xanthomonas campestris]
 emb|CDN20203.1| conserved hypothetical protein [Xanthomonas campestris pv. viticola]
Length=358

 Score = 39.7 bits (91),  Expect = 4.7, Method: Compositional matrix adjust.
 Identities = 52/221 (24%), Positives = 97/221 (44%), Gaps = 31/221 (14%)

Query  98   KHLSFPMT--SIELLETK----RHLFPCAQLSFPYDFDTDG----SLIDGIPDSLLSFTR  147
            K + F +T  S+ELL +K      + P  Q  + ++ D       SLI G+P   L F  
Sbjct  28   KRIEFYLTEYSVELLASKMSKGEFVVPAYQREYTWEHDRKSRFIESLILGLPIPFLFF--  85

Query  148  WERKIQLRKIVIASWQGRSWEPAARVLIRASEIFDLTQLEALEFSELPVGDTELELAENP  207
            WE      +IV  S + RS E      +R  E+  LT L   +FS+LP         E+ 
Sbjct  86   WEMPDGKLEIVDGSQRLRSIEEFVLGDLRLGELQSLTALSGFKFSDLP---------ESR  136

Query  208  KSSDLLEPLRRILLDSFGPKMQEL----KITTTSRLLLLLFLDSLPDLQRLSISADLANM  263
            +       +R I+L+    +        +I T S++       ++ +++R +++    ++
Sbjct  137  QRKINNRSIRGIVLNEHADEQARFDMFERINTGSKIA------NMAEVRRGALAGPFMDL  190

Query  264  ILNRTSYPRLESLQIMTDPDIEPSPIQEHPDLLFSRNDTLE  304
            I+  ++ P+  +L  ++  D+     +E     F+ +D LE
Sbjct  191  IIELSALPQFVALAPVSKKDLSEREHEELVARFFAYSDGLE  231


>ref|WP_033987660.1| hypothetical protein [Pseudomonas aeruginosa]
Length=358

 Score = 39.7 bits (91),  Expect = 4.9, Method: Compositional matrix adjust.
 Identities = 52/221 (24%), Positives = 96/221 (43%), Gaps = 31/221 (14%)

Query  98   KHLSFPMT--SIELLETK----RHLFPCAQLSFPYDFDTDG----SLIDGIPDSLLSFTR  147
            K + F +T  S+ELL  K      + P  Q  + ++ D       SLI G+P   L F  
Sbjct  28   KRIEFYLTEYSVELLANKMSKGEFVVPAYQREYTWEHDRKSRFIESLILGLPIPFLFF--  85

Query  148  WERKIQLRKIVIASWQGRSWEPAARVLIRASEIFDLTQLEALEFSELPVGDTELELAENP  207
            WE      +IV  S + RS E      +R  E+  LT L   +FS+LP         E+ 
Sbjct  86   WEMPNGKLEIVDGSQRLRSIEEFVLGDLRLGELQSLTALSGFKFSDLP---------ESR  136

Query  208  KSSDLLEPLRRILLDSFGPKMQEL----KITTTSRLLLLLFLDSLPDLQRLSISADLANM  263
            +       +R I+L+    +        +I T S++       ++ +++R +++    ++
Sbjct  137  QRKINNRSIRGIVLNEHADEQARFDMFERINTGSKIA------NMAEVRRGALAGPFMDL  190

Query  264  ILNRTSYPRLESLQIMTDPDIEPSPIQEHPDLLFSRNDTLE  304
            I+  +  P+  +L  ++  D++    +E     F+ +D LE
Sbjct  191  IIELSVLPKFVALAPVSKKDLDEREHEELVARFFAYSDGLE  231


>gb|EMS21313.1| hypothetical protein RHTO_01779 [Rhodosporidium toruloides NP11]
 emb|CDR49409.1| RHTO0S26e01134g1_1 [Rhodosporidium toruloides]
Length=470

 Score = 39.7 bits (91),  Expect = 6.2, Method: Compositional matrix adjust.
 Identities = 24/85 (28%), Positives = 40/85 (47%), Gaps = 9/85 (11%)

Query  7   IPTFIPIDIYREVIGQLFLDSISPNWLEREEIKDERRIKLFEDLLSCCLVSKEWLAITRP  66
           +PT +PI++ R+++  L   S++P         D+R+      L  CCL SK   A+ +P
Sbjct  1   MPTNLPIELVRQILVALGPCSLNP---------DDRQDATRRTLYCCCLASKALKAVAQP  51

Query  67  FLYLDIDLSCIDNLDALFNTLSLNP  91
            LY  + L     +    + L  NP
Sbjct  52  LLYQAVVLRRHTTVYKFSSALHANP  76


>ref|XP_001830880.2| hypothetical protein CC1G_02331 [Coprinopsis cinerea okayama7#130]
 gb|EAU90944.2| hypothetical protein CC1G_02331 [Coprinopsis cinerea okayama7#130]
Length=394

 Score = 39.3 bits (90),  Expect = 7.1, Method: Compositional matrix adjust.
 Identities = 22/63 (35%), Positives = 31/63 (49%), Gaps = 7/63 (11%)

Query  50   LLSCCLVSKEWLAITRPFLYLDIDLSCIDNLDALFNTLSLNPYIRPYVKHLSFPMTSIEL  109
            L +CCLVS++W A+ +P LY  I L        L   L  +P       HL+  +T + L
Sbjct  48   LRACCLVSQQWKALAQPLLYQTITLRSPSKFRGLLEALQSSP-------HLAAGVTRVVL  100

Query  110  LET  112
            L T
Sbjct  101  LGT  103


>ref|WP_009466141.1| hypothetical protein [Roseibium sp. TrichSKD4]
 gb|EFO33796.1| conserved hypothetical protein [Roseibium sp. TrichSKD4]
Length=291

 Score = 38.9 bits (89),  Expect = 7.9, Method: Compositional matrix adjust.
 Identities = 27/86 (31%), Positives = 44/86 (51%), Gaps = 2/86 (2%)

Query  162  WQGRSWEPAARVLIRASEIFDLTQLEALEFSELPVGDTELELAENPKSSDLLEPLRRILL  221
            +Q R   P  R+  R  E F   ++E++E     V   ++E+ + P  +DL+EPLR+ LL
Sbjct  181  YQERLLSPEVRIF-RVGEKFLAFRIESVELDYRSVDREKIEITQIPPPADLVEPLRK-LL  238

Query  222  DSFGPKMQELKITTTSRLLLLLFLDS  247
            D  G     +   T+S    L FL++
Sbjct  239  DRIGLNFSAVDFKTSSDTGTLCFLEA  264


>ref|WP_032830587.1| hypothetical protein [Pseudomonas sp. GM79]
Length=357

 Score = 38.9 bits (89),  Expect = 8.9, Method: Compositional matrix adjust.
 Identities = 62/253 (25%), Positives = 109/253 (43%), Gaps = 33/253 (13%)

Query  98   KHLSFPMT--SIELLETK----RHLFPCAQLSFPYDFDTDGSLID----GIPDSLLSFTR  147
            K + F +T  SIELL  K      + P  Q SF ++ D     I+    G+P   L F  
Sbjct  27   KRIEFYLTEYSIELLAAKVKNGEFVVPPYQRSFTWEDDRKSRFIESVLLGLPIPFLFF--  84

Query  148  WERKIQLRKIVIASWQGRSWEPAARVLIRASEIFDLTQLEALEFSELPVGDTELELAENP  207
            WE      +IV  S + R+         R  E+  LT+L +  F+ELP         E+ 
Sbjct  85   WEMPNGKLEIVDGSQRLRTIAEFVHDDFRLGELESLTKLSSFTFAELP---------ESR  135

Query  208  KSSDLLEPLRRILLDSFGPKMQEL----KITTTSRLLLLLFLDSLPDLQRLSISADLANM  263
            K       +R I+L+    +        +I T S++       +  +++R +++ D  ++
Sbjct  136  KRKINNRSIRGIVLNEHADEQARFDLFERINTGSKIA------NHAEVRRGALAGDFMSL  189

Query  264  ILNRTSYPRLESLQIMTDPDIEPSPIQEHPDLLFSRNDTLERLARAFPLLRVIGASPYMS  323
            I+     PRL++L  ++  D +    ++    LF+  D L+   +  P   + G    M+
Sbjct  190  IIELAVDPRLKTLAPVSTKDEKERGREDLVTRLFAYGDGLQGY-KDRPAEFLFGYVKGMN  248

Query  324  EQDIYADAELIPD  336
            E+ + AD  LI +
Sbjct  249  ER-VAADPNLIQE  260


>ref|XP_008040170.1| hypothetical protein TRAVEDRAFT_49159 [Trametes versicolor FP-101664 
SS1]
 gb|EIW56331.1| hypothetical protein TRAVEDRAFT_49159 [Trametes versicolor FP-101664 
SS1]
Length=265

 Score = 38.5 bits (88),  Expect = 9.0, Method: Compositional matrix adjust.
 Identities = 20/54 (37%), Positives = 31/54 (57%), Gaps = 0/54 (0%)

Query  47  FEDLLSCCLVSKEWLAITRPFLYLDIDLSCIDNLDALFNTLSLNPYIRPYVKHL  100
           F DL +C L  ++W++ +R  LY  I L     LDAL NT++  P++   V+ L
Sbjct  31  FVDLCACALTCRDWVSRSRCNLYSSIKLRNARQLDALLNTIASLPFLADLVQEL  84


Lambda      K        H        a         alpha
   0.322    0.139    0.412    0.792     4.96 

Gapped
Lambda      K        H        a         alpha    sigma
   0.267   0.0410    0.140     1.90     42.6     43.6 

Effective search space used: 3927647407840


  Database: nr
    Posted date:  Sep 23, 2015 12:05 AM
  Number of letters in database: 26,053,659,533
  Number of sequences in database:  71,551,133


Matrix: BLOSUM62
Gap Penalties: Existence: 11, Extension: 1
Neighboring words threshold: 11
Window for multiple hits: 40
```
